# Risk factors and outcome of patients with eclampsia at a tertiary hospital in Egypt

**DOI:** 10.1186/s12884-017-1619-7

**Published:** 2017-12-22

**Authors:** Ahmad Mahran, Hashem Fares, Reham Elkhateeb, Mahmoud Ibrahim, Haitham Bahaa, Ahmad Sanad, Alaa Gamal, Mohamed Zeeneldin, Eissa Khalifa, Ahmed Abdelghany

**Affiliations:** Department of Obstetrics and Gynecology, Faculty of Medicine, Minia University, Minia, Egypt

**Keywords:** Eclampsia, maternal mortality, maternal morbidity, perinatal outcome

## Abstract

**Background:**

Eclampsia is a major cause of maternal and neonatal morbidity and mortality in low and middle income countries. The aim of this study was to assess the risk factors and maternal and perinatal outcome in patients with eclampsia in order to get reliable data that helps in reducing the incidence and improving the outcome in an area with high incidence of eclampsia.

**Methods:**

Retrospective study including 250 patients diagnosed with eclampsia at Minia Maternity University Hopsital, Minia, Egypt in the period between January 2013 and December 2014.We analyzed the data obtained from medical records of these patients including patient characteristics, medical, obstetric, current pregnancy history, data on hospital admission, treatment given at hospital and maternal and perinatal outcome. Statistical analysis was done using SPSS version 21.

**Results:**

During the study period, 21690 women gave birth in the hospital; of which 250 cases of eclampsia were diagnosed (1.2%).Four women died (case fatality rate 1.6%). The main risk factors identified were young age, nulliparity, low level of education, poor ante-natal attendance and pre-existing medical problems. The most common complication was HELLP syndrome (15.6%). Magnesium sulphate therapy was given to all patients but there was lack of parenteral anti-hypertensive therapy. Forty six cases delivered vaginally (18.4%). Assisted delivery was performed in 22 (8.8%) cases and caesarean section in 177 (70.8%) cases; 151(60.4%) primary caesarean sections and 26 (10.4%) intra-partum. Perinatal deaths occurred in 11.9% on cases. Prematurity and poor neonatal services were the main cause.

**Conclusion:**

Morbidity and mortality from eclampsia are high in our setting. Improving ante-natal and emergency obstetric and neonatal care is mandatory to improve the outcome.

## Background

Hypertensive disorders (HD) are the most common medical disorders complicating pregnancy with overall prevalence between 5 -10% and are considered as a main cause for maternal and perinatal morbidity and mortality worldwide [1, 2]. Eclampsia is defined as occurrence of convulsions in association with pre-eclampsia and is a serious life threatening complication of HD of pregnancy [3].

The incidence and morbidities associated with eclampsia varies greatly between developed and developing countries. Global and regional estimates indicated a crude incidence of eclampsia fluctuating from 0 to 0.1 in Europe and up to 4% in Nigeria [4, 5]. The case fatality rate (number of deaths/number of cases) of eclampsia ranges from 0-1.8% in high-income countries up to 17.7% in India [6]. These data highlights the impact of the socioeconomic standard and availability of medical facilities on the magnitude of the problem.

In Egypt; where this study was conducted, HD of pregnancy account for 14.9% of causes of maternal mortality [7]. However, there are no clear records estimating the exact incidence and morbidities and mortalities associated with eclampsia.

The aim of this study is to assess the risk factors and to study the maternal and perinatal outcome of in eclamptic patients at a tertiary hospital in Minia, Egypt as one of the low resource countries.

## Methods

This study was a retrospective study conducted at Minia Maternity University Hospital in Minia, Egypt. This hospital serves a catchment area of approximately 600.000 people and receives referred cases from other central and district hospitals in Minia governorate that has a population of 5.156.702 people according to the last official estimates in 2015.The annual number of deliveries at this tertiary hospital ranges from10.000 to 12.000.As the referral system is not well established, many cases are self-referred from home or private health centers without enough data. Most of these cases are complicated cases which add more challenge to the care givers at the hospital.

Medical records of all patients diagnosed with eclampsia in the period between January 2013 and December 2014 were retrieved. Data were analyzed including demographic data, clinical presentation, Investigations, medical treatment received, method and outcome of delivery, maternal and perinatal outcome and discharge data. Data was filled in data collection sheets anonymously. Medical records of all patients who gave birth at the hospital at the same time period were retrieved and their demographic data were filled in data collection sheets for comparison.

### Inclusion criteria

Eclampsia defined as reliable history of seizures at home, on the way to the hospital or inside the hospital in association with high blood pressure and proteinuria.

### Exclusion criteria

All cases with epilepsy, encephalopathy, tetanus, meningitis, hypoglycemia, ketoacidosis pyrexia and suspected drug toxicity were excluded. These cases were included in the all deliveries group.

### Statistical analysis

Statistical analysis was performed using the Statistical Package for Social Science (SPSS Inc, Chicago) version 21 for Microsoft Windows. Descriptive statistics were used to examine the distribution of patients' characteristics and the outcomes. A single logistic regression model was applied to estimate the relationship between the risk factors as independent variables and eclampsia as the outcome. A P value <0.05 was considered significant.

### Ethical statement

This study was approved by the ethical committee of the Department of Obstetrics and Gynecology, Minia University Hospital on 15/03/2015 (Registration number: MUH15323). As the study was retrospective, Obtaining consent forms from patients was not applicable.

## Results

During the study period, 21690 women gave birth at Minia Maternity Hospital. Two hundred and fifty patients were diagnosed with eclampsia (1.2%). The Characteristics and risk factors in these patients are shown in Table 1.

**Table 1:** Risk factors in the study population

Risk factor	Deliveries	Eclampsia	Adjusted Odds ratio (95% CI)	*P* value
Age:				
< 20 years	3732 (17.2%)	106 (42.4%)	2.05(1.95-2.28)	<0.001
20-29 years	12421 (57.3%)	89 (35.6%)	Reference	
30-34 years	4107 (18.9%)	38 (15.2%)	1.29(1.18-1.39)	0.185
>35 years	1430 (6.6%)	17 (6.8%)	1.67(1.59-1.71)	0.05
Parity:				
Nullipara	7965 (36.7%)	193 (77.2%)	3.59(3.03-4.01)	<0.001
Multipara	13725 (63.3%)	57 (22.8%)	Reference	
BMI				
<20	1483 (6.8%)	27 (10.8%)	0.84(0.73-0.95)	0.59
20 to <25	5013 (23.2%)	52 (20.8%)	Reference	
25 to <30	7531 (34.7%)	41 (16.4%)	0.52(0.45-0.63)	0.002
30 to <35	6423 (29.6%)	73 (29.2%)	1.26(1.15-1.38)	0.187
≥35	1240 (5.7%)	57 (22.8%)	4.59(4.14-4.93)	<0.001
Duration of marriage before pregnancy (primigavida)				
<6 months	2547 (32%)	127 (65.8%)	3.05(2.73-3.59)	<0.001
6-12 months	4221 (53%)	54 (28%)	Reference	
>12 months	1197 (15%)	12 (6.2%	0.75(0.62-0.87)	0.442
Change of partner(second marriage) (multipara)	221 (1.6%)	11 (19.3%)	7.63(6.82-8.96)	<0.001
Interval between pregnancies(multipara)				
<5 years	5913 (74.2%)	14 (24.6%)	Reference	
5-10 years	1340 (16.8%)	18 (31.6%)	2.74(2.25-3.36)	<0.001
>10 years	712 (8.9%)	25 (43.9%)	7.33(6.63-8.34)	<0.001
Level of education				
-None	2378 (11%)	165 (66%)	8.24(7.15-9.53)	<0.001
-Primary	7423 (34.2%)	62 (24.8%)	2.42(2.28-2.54)	<0.001
-Secondary	8537 (39.4%)	21 (8.4%)	Reference	
-Tertiary	3352 (15.4%)	2 (0.8%)	0.24(0.16-.33)	0.055
Family history of preeclampsia				
Mother	173 (0.8%)	17 (6.8%)	3.07(2.92-3.18)	<0.001
sister	194 (0.9%)	19 (7.6%)	3.11(2.99-3.22)	<0.001
Pre-existing medical problems:				
-Pre-existing hypertension	198 (0.9%)	54 (21.6%)	8.12(7.09-9.15)	<0.001
-Pre-existing diabetes mellitus	194 (0.9%)	39 (15.6%)	5.63(4.59-6.65)	<0.001
-Pre-existing renal disease.	47 (0.2%)	7 (2.8%)	2.59(1.92-3.15)	<0.001
-Cardiac disease.	325 (1.5%)	4 (1.4%)	1.08(0.98-1.18)	0.876
-Anaemia	1865 (8.6%)	79 (31.6%)	2.53(2.01-2.92)	<0.001
- Known thrombophilia	75 (0.4%)	11 (4.4%)	2.54(2.09-3.04)	<0.001
Preeclampsia or PIH in previous pregnancy (multipara)	887 (11.1%)	34 (59.6%)	2.69(2.26-3.04)	<0.001
Multiple pregnancy	453(2.1%)	10(4%)	1.15(1.03-1.25)	0.041
Antenatal care visits:				
0	1041 (4.8%)	75 (30%)	4.38(3.43-5.39)	<0.001
1-3 visits	13623(62.8%)	161 (64.4%)	1.29(1.18-1.39)	0.534
4-8 visits	5769 (26.6%)	13 (5.2%)	Reference	
>8 visits	1257 (5.8%)	1 (0.4%)	0.35(0.27-0.43)	0.015

*PIH* pregnancy induced hypertension

Among 250 patients diagnosed with eclampsia, 148 patients had the seizures antenatally, 2 patients had the seizures intra-partum and 100 patients had the seizures after delivery. On admission, 154 patients (61.6%) had severe hypertension (diastolic BP≥110mmHg), 92 patients (36.8%) had mild hypertension (diastolic BP 90-<110mmHg), and 12 patients (4.8%) had a diastolic BP <90 mmHg The different presentation of cases on admission is shown in Table 2.

**Table 2:** The presenting symptoms and signs of cases on admission

	Frequency	Percentage
Seizures	42	16.8%
Headache	67	26.8%
Visual disturbances	23	9.2%
Labour pain	22	8.8%
Proteinuria	241	96.4%
Jaundice	14	5.6%
Vaginal bleeding	5	2%
Oedema	56	22.4%

Of 250 women, 22 cases presented in labour, 77 cases had induction of labour and 151 cases delivered with primary caesarean section. The onset and mode of deliveries in the study population are shown in Table 3.

**Table 3:** Onset and mode of delivery in the study population

	Number	Percentage
Onset of labour:		
-Spontaneous	22	8.8%
-Induced labour	77	30.8%
-Primary caesarean section	151	60.4%
Mode of delivery:		
-Vaginal delivery	51	20.4%
-Instrumental delivery	22	8.8%
-Intrapartum caesarean section:		
Caesarean after spontaneous labour	6	2.4%
Caesarean after induced labour	20	8%

Four maternal deaths occurred in the patients; two cases due to massive intracranial hemorrhage, one case due to HELLP syndrome, and one case due to intractable postpartum hemorrhage. Case fatality rate (CFR) was 1.6%. Maternal morbidities and mortalities are shown in Table 4.

**Table 4:** Maternal complications in patients with eclampsia

	Number	Percentage
Placental abruption	7	4.6%
Disseminated intravascular coagulopathy (DIC)	19	7.6%
Postpatum hemorrhage	24	9.6%
Intracranial hemorrhage	2	0.8%
HELLP syndrome	39	15.6%
Acute pulmonary oedema	2	0.8%
Renal impairment	18	7.2%
Liver impairment	26	10.4%
Anesthetic complications	6	2.4%
Massive blood transfusion	9	3.6%
Septic complications	4	1.6%
Maternal death	4	1.6%

The main medical treatments given to patients were magnesium sulphate and antihypertensive medications. Magnesium sulphate was given to all patients with eclampsia. There was lack of parenteral anti-hypertensive therapy. Oral anti-hypertensive treatment was given to 155 patients before delivery. The given medications included oral nifidepine and labetalol as shown in Table 5.

**Table 5:** Medications received by the study population

	Number	Percentage
Magnesium sulphate	250	100%
Anti-hypertensive therapy:		
-Oral nifidepine	155	62%
-Oral labetalol	23	9.2%

Of 250 women, nine (3.6%) had twins and one (0.4%) had triplet resulting in 261 foetuses. The perinatal outcome in the study population is shown in Table 6.

**Table 6:** Perinatal outcome in eclamptic patients

	Number	Percentage
Gestational age on admission:		
21-28 weeks	4	1.5%
28-34 weeks	112	42.9%
>34 weeks	145	55.6%
Birth weight (gram):		
≤500	1	0.4%
500-750	3	1.2%
750-1000	12	4.6%
1000-1500	59	22.6%
1500-2500	99	37.9%
>2500	87	33.3%
Apgar score at 5 minutes:		
0	7	2.7%
1-2	10	3.8%
3-6	14	5.4%
≥7	230	88.1%
Neonatal outcome:		
Stillbirth	7	2.7%
Early neonatal death	24	9.2%
NICU admission	49	18.8%
Hospital discharge	181	69.3%

*NICU* Neonatal intensive care unit

## Discussion

This study analyzes the risk factors and maternal and perinatal outcome in patients with eclampsia treated at Minia Maternity University Hospital in the period from January 2013 to December 2015. The percentage of patients with eclampsia in this study was 1.2% which is much higher than European and Gulf countries Kullberg [8–11] while lower than the incidence reported in Asian and sub-Saharan African countries [12–15]. This difference is obviously attributed to the variation in socio-economic level and standards of ante-natal care.

The main risk factors identified in this study were shown in Table 1. Maternal age was identified as a major risk factor with 106 patients (42.2%) was < 20 years. However, in the locality where the study was conducted, this reflects young age at marriage which is associated with low educational and economic standards and consequently poor antenatal attendance which can be a major confounder.

Nulliparity is a well-known risk factor for eclampsia [16, 17]. In the current study 77.2% of patients were nullipara. 68.4% of patients had a BMI ≥ 25 and 22.8% had a BMI ≥ 35; a finding that strengthens the role of high BMI in the pathogenesis of pre-eclampsia mostly through placental vasculopathy and endothelial dysfunction [18, 19]. Family history of pre-eclampsia was noted in 14.4% of patients (6.8% in mother, 7.6% in sister). Family history of pre-eclampsia is known to triple the risk of developing pre-sclampsia [16].

In this study, the short duration of marriage was found to increase the risk of eclampsia with 127 patients (65.8%) getting pregnant within 6 months of marriage. The reason for this is not clear. However, Yousefi et al., suggested that the length of sperm exposure affects the risk of preeclampsia which might me a reasonable explanation [20]**.**

Low socioeconomic standard in the area where the study was conducted obviously affected the incidence of eclampsia. That was reflected on the educational level and the frequency of ante-natal visits. 66.6% of patients had no education at all and 24.8% received primary education. On the other hand, only two patients (0.8%) were educated up to the university level. Seventy-five patients (30%) did not attend ante-natal visits at all through pregnancy and 161 (64.4%) patients had less than 4 ante-natal visits. Only one patient had more than 8 ante-natal visits developed eclampsia. This patient had pre-existing hypertension and diabetes mellitus with renal impairment. Both educational level and poor ante-natal attendance were identified as risk factors for pre-eclampsia /eclampsia in a recent WHO secondary analysis in low and middle income countries [21].

Pre-existing medical problems were identified in a significant number of patients. Pre-existing hypertension was found in 54 (21.6%) cases, pre-existing diabetes mellitus in 39(15.6%) cases, pre-existing renal disease in 7 (2.8%) cases and known thrombophilia in 11 (4.4%) cases. This is most properly due to the associated vasculopathy in these disorders, which plays a major role in the pathogenesis of pre-eclampsia [16, 17]. Anemia was identified in 79 (31.6%) of cases; a finding that is worse evaluation in further prospective studies to assess for a possible association and the effect of ante-natal treatment of anemic patients on their risk of developing pre-eclampsia/eclampsia.

In the patients who were multipara, additional risk factors were identified. New marriage was noted in 11/57 (19.3%) patients. Interval of 5-10 years since previous pregnancy was noted in 18/57 (31.6%) patients and interval of more than 10 years in 25/57(43.9%) patients. The probability of pre-eclampsia is increased for each year increase in the interval since previous pregnancy [16, 17]. Previous history of preeclampsia or PIH was identified in 34/57 (59.6%) cases. These findings are in agreement with other studies in other regional distributions [11, 22, 23].

Twenty-two patients (8.8%) were presented in labour, while 77 (30.8%) patients had induction of labour and 151 (60.4%) patients had primary caesarean section. Intraparum caesarean section was performed for 6/22 (27.3%) of those who presented in labour and 20/77 (26%) of those who had induction of labour giving a total number of caesarean section of 177/250 (70.8%). This high rate of caesarean section might have many explanations including lack of monitoring facilities during vaginal delivery which obviously takes longer time in addition to lack of parenteral anti-hypertensive therapy which make the doctors anxious about monitoring of patients with uncontrollable blood pressure during vaginal delivery. Inadequate fetal monitoring equipment and lack of fetal scalp sampling in the hospital where the study was conducted is responsible for low threshold of performing caesarean section for patients in labour. Previous studies had shown that caesarean delivery is associated with higher rate of complications in cases of preeclampsia [24–27]. Further studies assessing the effect of mode of delivery on the maternal and perinatal outcome in the same area are needed.

The most common complications of eclampsia identified in this study were HELLP syndrome 39/250 (15.6%), Liver impairment 26/250 (10.6%), postpartum hemorrhage 24/250 (9.6%), disseminated intravascular coagulopathy 19/250 (7.6%) and renal impairment 18/250 (7.2%). There were two cases of intracranial hemorrhage. Four cases of sepsis were identified; all of them occurred in cases delivered by caesarean section. These findings differ from studies done in Saudi Arabia and Kuwait, which identified abruption placenta as the most common complications [10, 11]. The high incidence of hepatic complications in the study population highlights the importance of studying the antenatal liver abnormalities in the pregnant population and its impact on pregnancy outcome particularly in a region with high prevalence of hepatitis infection. There were four cases of maternal mortalities in the study population; two cases due to massive intracranial hemorrhage, one case due to HELLP syndrome and one case due to intractable postpartum hemorrhage. The case fatality rate was 1.6%, which is lower than the rates quoted in Nigeria (6.5%) and Tanzania (11%) but higher than the rates quoted in Saudi Arabia, Kuwait, UK and Sweden (0%) [8–11, 14, 15, 28].

Magnesium sulphate therapy was administered in all patients as per hospital guidelines that reflect availability and orientation with its role in preventing further seizures. However, in many occasions, MgSO4 was not available at the hospital and there was lag of time till it could be made available and be given to patients presented with severe preeclampsia. As a result, many patients have seizures during this time lag. In addition, we do not have sufficient data about whether the proper dose was given after delivery. Infusion pump are not available at the hospital, so we depend on clinical monitoring by the nursing staff. We have high work load at the hospital and relatively insufficient nursing staff, so improper dosing or early discontinuation of treatment are likely. Oral anti-hypertensive therapy was given in 178 of 250 women to control blood pressure at the hospital. More aggressive oral therapy should have been administered in some cases till a reliable supply of parenteral antihypertensive therapy could be made available. However, oral antihypertensive drugs could not be started in some cases due to disturbed conscious level. The suboptimal blood pressure control is previously identified as possible cause of complications in women with severe hypertension [29, 30] and it might be a major cause for complications in this study.

One hundred and ninety four women (77.6%) had seizure before admission. However, many patients were presented with severe hypertension and developed seizures before starting MgSo4 and anti-hypertensive therapy. This is another point related to our limited resources. As explained before, in many occasions, MgSO4 was not available at the hospital and there was lag of time till it could be made available. As a result, many patients have seizures during this time lag. In addition, improper dosing and early discontinuation of MgSO4 and antihypertensive therapy are likely due to high work load and relatively insufficient nursing staff. Of the 100 seizures after delivery, 73 patients developed postpartum seizures before admission in our hospital and 27 patients developed seizures after admission. That can be due to delay in the use of MgSO4 and improper control of BP. Therefore, aggressive interventions to improve care are warranted**.**

There were 7 cases of stillbirth; 5 ante-natal and 2 intra-partum deaths and 24 cases of early neonatal deaths giving a perinatal mortality rate (PNMR) of 11.9% which is a higher rate as compared with that quoted from studies in Europe and Gulf countries [8–11]. Studies assessing PNMR in preeclampsia and eclampsia in Latin America identified a rate of 3.26% [21]. Studies in African countries identified varying rates from 11.39% in Algeria to 22.11% in DRC [21]. PNMR from eclampsia only was 30% in Tanzania [14]. In Asian countries with low or middle income, the PNMR in pre-eclapmsia and eclampsia varies from 1.3% in Vietnam to 15.76 in Nepal [21]. Most of perinatal deaths in the current study were in premature babies. The inadequate treatment of preterm neonates and insufficient NICU are the major contributing factors to these deaths.

The strength of this study was the large number of women included, extraction of data from hospital medical records and providing data for region with high incidence which can help to reduce incidence and improve outcome. The limitation of the study was the referral bias as many of the patients with eclampsia were referred from outside the hospital catchment area which did not allow accurate estimation of the incidence of eclampsia.

## Conclusions

In our setting, eclampsia remains a major risk to maternal and neonatal lives. The low socio-economic standards, low level of education and poor ante-natal care were identified as major risk factors. PNMR was high and mainly attributed to prematurity. Health policies need to be implemented to provide better ante-natal care with more orientation about the importance of early detection of cases with high blood pressure during pregnancy to avoid development of complications. Better Education and training of health workers is mandatory to improve care of critical cases. Making the parental antihypertensive therapy available and improving neonatal services can reduce the rate of maternal and neonatal complications. Further studies are needed to assess the influence of mode of delivery on maternal and neonatal outcome in cases with severe pre-eclampsia and eclampsia.

## Abbreviation

CFR: Case fatality rate
DIC: Disseminated intravascular coagulopathy
HD: Hypertensive disorders
NICU: Neonatal intensive care unit
PIH: Pregnancy induced hypertension
PNMR: Perinatal mortality rate
SPSS: Statistical Package for Social Sciences
